# GLP-1 Limits Adipocyte Inflammation and Its Low Circulating Pre-Operative Concentrations Predict Worse Type 2 Diabetes Remission after Bariatric Surgery in Obese Patients

**DOI:** 10.3390/jcm8040479

**Published:** 2019-04-09

**Authors:** Maitane Izaguirre, Javier Gómez-Ambrosi, Amaia Rodríguez, Beatriz Ramírez, Sara Becerril, Víctor Valentí, Rafael Moncada, Xabier Unamuno, Camilo Silva, Magdalena de la Higuera, Javier Salvador, Ignacio Monreal, Gema Frühbeck, Victoria Catalán

**Affiliations:** 1 Metabolic Research Laboratory, Clínica Universidad de Navarra, 31008 Pamplona, Spain; mizaguirrea@unav.es (M.I.); jagomez@unav.es (J.G.-A.); arodmur@unav.es (A.R.); bearamirez@unav.es (B.R.); sbecmac@unav.es (S.B.); xunamuno@unav.es (X.U.); 2 CIBER Fisiopatología de la Obesidad y Nutrición (CIBEROBN), Instituto de Salud Carlos III, 31008 Pamplona, Spain; vvalenti@unav.es (V.V.); rmoncada@unav.es (R.M.); csilvafr@unav.es (C.S.); jsalvador@unav.es (J.S.); 3 Obesity and Adipobiology Group, Instituto de Investigación Sanitaria de Navarra (IdiSNA), 31008 Pamplona, Spain; 4 Department of Surgery, Clínica Universidad de Navarra, 31008 Pamplona, Spain; 5 Department of Anesthesia, Clínica Universidad de Navarra, 31008 Pamplona, Spain; 6 Department of Endocrinology & Nutrition, Clínica Universidad de Navarra, 31008 Pamplona/Madrid, Spain; mhiguera@unav.es; 7 Department of Biochemistry, Clínica Universidad de Navarra, 31008 Pamplona, Spain; jimonreal@unav.es

**Keywords:** GLP-1, inflammation, obesity, adipose tissue, bariatric surgery, T2D remission

## Abstract

Objective: Glucagon-like peptide (GLP)-1 has been proposed as a key candidate in glucose improvements after bariatric surgery. Our aim was to explore the role of GLP-1 in surgically-induced type 2 diabetes (T2D) improvement and its capacity to regulate human adipocyte inflammation. Methods: Basal circulating concentrations of GLP-1 as well as during an oral glucose tolerance test (OGTT) were measured in lean and obese volunteers with and without T2D (*n* = 93). In addition, GLP-1 levels were determined before and after weight loss achieved by Roux-en-Y gastric bypass (RYGB) (*n* = 77). The impact of GLP-1 on inflammation signalling pathways was also evaluated. Results: We show that the reduced (*p* < 0.05) circulating levels of GLP-1 in obese T2D patients increased (*p* < 0.05) after RYGB. The area under the curve was significantly lower in obese patients with (*p* < 0.01) and without (*p* < 0.05) T2D compared to lean volunteers while obese patients with T2D exhibited decreased GLP-1 levels at baseline (*p* < 0.05) and 120 min (*p* < 0.01) after the OGTT. Importantly, higher (*p* < 0.05) pre-operative GLP-1 concentrations were found in patients with T2D remission after RYGB. We also revealed that exendin-4, a GLP-1 agonist, downregulated the expression of inflammation-related genes (*IL1B*, *IL6*, *IL8*, *TNF*) and, conversely, upregulated the mRNA levels of *ADIPOQ* in human visceral adipocytes. Furthermore, exendin-4 blocked (*p* < 0.05) LPS-induced inflammation in human adipocytes via downregulating the expression and secretion of key inflammatory markers. Conclusions: Our data indicate that GLP-1 may contribute to glycemic control and exert a role in T2D remission after RYGB. GLP-1 is also involved in limiting inflammation in human visceral adipocytes.

## 1. Introduction

Obesity is increasing worldwide, reaching epidemic proportions and becoming an international public health challenge [1]. These data are alarming because epidemiological studies have identified obesity as the key in a growing number of chronic diseases. Emerging evidence reveals that sustained obesity as well as its associated chronic and unresolved inflammatory state promote an imbalance of adaptive homeostatic mechanisms, leading to adipose tissue dysfunction and the development of its related comorbidities [2]. In spite of relevant efforts to develop effective pharmacological treatments, bariatric surgery remains the most effective option for achieving important and sustained weight loss [3]. Furthermore, this procedure also exhibits favourable outcomes in the resolution of obesity-associated comorbidities including type 2 diabetes (T2D) [3,4]. The mechanisms underlying metabolic benefits are complex and involve the combination of multiple neuroendocrine signals as well as anatomical, endocrine and metabolic effects [3]. In this regard, recent trials evaluating the gut hormone glucagon-like peptide-1 (GLP-1) as well as specific GLP-1 receptor (GLP-1R) agonists for the treatment of T2D alone or T2D and obesity have shown promising results [5,6,7].

GLP-1 is an incretin synthesized in and secreted from enteroendocrine L-cells in the ileum and colon in response to nutrient ingestion, particularly fats and carbohydrates [8]. GLP-1 has a wide array of physiological actions but its main roles include the stimulation of insulin secretion and the suppression of glucagon release in a glucose-dependent manner as well as the inhibition of gastric emptying and food intake, leading to a reduction of glycaemia and weight loss [8]. Since native GLP-1 is immediately inactivated by the ubiquitous serine protease dipeptidyl peptidase enzyme (DPP)-4, degradation-resistant analogs of GLP-1 (exendin-4, liraglutide, semaglutide, dulaglutide, albiglutide or lixisesnatide) have been developed and approved for use as second or third-line therapy in the treatment of T2D [9]. Among these, exendin-4, a short incretin-mimetic peptide with an approximately 53% identity with native GLP-1 is a potent agonist for the GLP-1R and promotes insulinotropic effects enhancing the physiological functions of β-cells and lowering plasma glucose levels [10].

Since bariatric surgery induces weight loss and T2D remission together with a rise in postprandial GLP-1 secretion, the role of GLP-1 in the response to surgery-associated benefits has been extensively studied [4,11]. In this sense, the response to the administration of GLP-1 agonists has been shown to predict the efficacy of Roux-en-Y gastric bypass (RYGB) on glucose tolerance in diet-induced obese rats [12]. However, the degree to which GLP-1 is responsible for the metabolic success after bariatric surgery is continually debated [6] with studies suggesting that GLP-1 is one of the best predictors of T2D remission after RYBG [13], and other findings showing that the increased GLP-1 secretion is not enough to maintain normal glucose tolerance [14].

In addition to the well-documented glucoregulatory actions of GLP-1, its role as an anti-inflammatory factor on different models has been reported [15,16]. Importantly, GLP-1 administration improved the endothelial dysfunction, inflammation and oxidative stress in patients with T2D [17]. Moreover, different studies have revealed that GLP-1 agonists reduced the circulating levels of pro-inflammatory C-reactive protein (CRP) [18] and interleukin (IL)-6 [19] in patients with T2D. However, it remains unclear whether GLP-1 modulates inflammation directly by interacting with its receptor expressed in circulating immune cells or indirectly by promoting weight loss and glycaemic improvement [20].

Collectively, these observations suggest that beyond weight loss, bariatric surgery improves the resolution of T2D increasing the levels of GLP-1 [3]. In this regard, we tested the hypothesis that pre-operative GLP-1 levels vary among groups of patients with different outcomes of T2D after RYGB, and the GLP-1 response differs during a 2-h oral glucose tolerance test (OGTT) between control subjects and obese patients with and without T2D. According to the additional and beneficial role of GLP-1 in regulating inflammation, the effects of exendin-4 in the regulation of the inflammatory response on human adipocytes was further explored. Finally, we also studied the role of exendin-4 in LPS-mediated inflammation signalling pathways.

## 2. Material and Methods

### 2.1. Patient Selection

Samples from 93 subjects (40 males and 53 females) recruited from healthy volunteers and patients attending the Departments of Endocrinology and Nutrition and Surgery at the Clínica Universidad de Navarra were used to analyse the effect of obesity and T2D on the plasma levels of GLP-1. Volunteers underwent a clinical assessment including medical history, physical examination, body composition analysis and co-morbidity evaluation by a multidisciplinary consultation team. Body mass index (BMI) was calculated as weight in kilograms divided by the square of height in meters and body fat (BF) was estimated by air-displacement-plethysmography (Bod-Pod^®^, Life Measurements, Concord, CA, USA). Obese patients were further subclassified into two groups ((normoglycaemia (NG) or T2D)) following the criteria of the Expert Committee on the Diagnosis and Classification of Diabetes [21]. In addition, a group of 77 patients with obesity (28 males and 49 females) was selected to investigate the effect of weight loss on circulating GLP-1 levels 1 year after RYGB. Remission of T2D in obese patients was evaluated 3 years after RYGB and was defined according to the American Diabetes Association criteria [21]. Specifically, remission was defined by (glycosylated hemoglobin) HbA1c <6.0%, fasting glucose <100 mg/dL, and no use of antidiabetic medication for at least 12 months. The novel scoring system (DiaRem score) to predict the probability of remission of T2D after RYGB was also calculated based on age, pre-operative HbA1c as well as the use of metformin, sulfonylurea, glitazones, and/or insulin, as previously reported [22].

Visceral adipose tissue (VAT) samples were also collected from patients undergoing either Nissen fundoplication (for hiatus hernia repair in lean volunteers) or RYGB (for surgical treatment of severe obesity) at the Clínica Universidad de Navarra. Both interventions were carried out via a laparoscopic approach. Tissue samples were immediately frozen in liquid nitrogen and stored at −80 °C for subsequent analyses. The study was approved, from an ethical and scientific standpoint, by the Hospital’s Ethical Committee responsible for research and the written informed consent of participants was obtained.

### 2.2. Analytical Procedures

Plasma samples were obtained by venipuncture after an overnight fast to avoid potential confounding influences due to hormonal rhythmicity. Glucose was analysed by an automated analyser (Roche Cobas 8000, Roche, Basel, Switzerland). Insulin was measured by means of an enzyme-amplified chemiluminescence assay (IMMULITE^®^, Diagnostic Products Corp., Los Angeles, CA, USA) with intra- and inter-assay coefficients of variation of 4.2 and 5.7%, respectively. Insulin sensitivity and resistance were calculated using the QUICKI and HOMA indices, respectively. Total cholesterol, high-density lipoprotein (HDL-cholesterol) and low-density lipoprotein (LDL-cholesterol) levels were calculated as previously described [23]. Uric acid, creatinine, alanine aminotransferase (ALT), aspartate aminotransferase (AST), alkaline phosphatase (ALP) and γ-glutamyltransferase (γ-GT) were measured by enzymatic tests in an automated analyser (Roche/Hitachi Modular P800). High-sensitivity CRP, fibrinogen and von Willebrand factor antigen (vWF) concentrations were determined as previously reported [23]. Leptin was measured by a double-antibody RIA method (Linco Research, Inc., St. Charles, MO, USA) and intra- and inter-assay coefficients of variation were 5.0 and 4.5%, respectively. Adiponectin (Biovendor, Brno, Czech Republic) and GLP-1 (Alpco, Salem, MA, USA) were quantified by commercially available enzyme-linked immunesorbent assay (ELISA) kits with intra- and inter-assay coefficients of variation being 3.9% and 6.0%, respectively, for the former, and 4.2% and 7.8% for the latter. IL-1β, IL-6, IL-8 and monocyte chemoattractant protein (MCP)-1 circulating levels were determined by commercially available ELISA kits (RayBiotech, Inc., Norcross, GA, USA) before and after RYGB. The intra- and inter-assay coefficients of variation were <10 and <12%, respectively, for all analysed molecules.

### 2.3. Oral Glucose Tolerance Test

After an overnight fast, subjects underwent a standard OGTT ingesting 75 g glucose dissolved in water. Blood samples were collected from a patent venous catheter at baseline and at time points 30, 60, 90 and 120 min from glucose intake. Blood samples for determination of GLP-1 were collected in vacutainer tubes containing a protease inhibitor cocktail including DPP-4 inhibitors (P800, Becton Dickinson, Franklin Lakes, NJ, USA). Blood samples for determination of glucose and insulin were collected in EDTA-containing vacutainer tubes (Becton Dickinson, Franklin Lakes, NJ, USA). Tubes were immediately placed on ice and centrifuged at 4 °C for 15 min at 1500× *g*. Plasma samples were stored at −80 °C for subsequent analyses.

### 2.4. RNA Extraction and Real-Time PCR

RNA extraction from VAT and adipocytes was performed by homogenization with an Ultra-Turrax^®^ T25 basic (IKA^®^ Werke Gmbh, Staugen, Germany) using QIAzol^®^ Reagent (Qiagen, Hilden, Germany) and was purified using the RNeasy Mini Lipid Kit (Qiagen). All samples were treated with DNase I (RNase Free DNase set, Qiagen). The RNA integrity and quantification were performed with the NanoPhotometer (Implen GmbH, München, Germany). For first strand cDNA synthesis constant amounts of 3 µg of total RNA were reverse transcribed in a 60 µL final volume using random hexamers (Roche) as primers and 300 units of M-MLV reverse transcriptase (Invitrogen, Carlsbad, CA, USA). Gene transcript levels were quantified by Real-Time PCR (7300 Real Time PCR System, Applied Biosystem, Foster City, CA, USA) as previously described [24]. Primers and probes (Supplementary Materials, Table S1) were designed using the software Primer Express 2.0 (Applied Biosystems, Foster City, CA, USA). The *18S* rRNA (Applied Biosystems) was the endogenous control gene for Real-Time PCR experiments and relative quantification was calculated using the ΔΔCt formula. Relative mRNA expression was expressed as fold expression over the calibrator sample (average of gene expression corresponding to the lean or unstimulated group).

### 2.5. Adipocyte Culture

Human stroma-vascular fraction cells (SVFC) were isolated from VAT from subjects with obesity as previously described [25]. SVFC were seeded at 2 × 10^5^ cells/well and grown in adipocyte medium (DMEM/F-12 (1:1)) (Invitrogen), 17.5 mol/L glucose, 16 μmol/L biotin, 18 μmol/L panthotenate, 100 μmol/L ascorbate and antibiotic-antimycotic] supplemented with 10% newborn calf serum (NCS). After 4 days, the medium was changed to adipocyte medium supplemented with 3% NCS, 0.5 mmol/L 3-isobutyl-1-methylxanthine (IBMX), 0.1 μmol/L dexamethasone, 1 μmol/L BRL49653 and 10 μg/mL insulin. After a 3-day induction period, cells were fed every 2 days with the same medium but without IBMX and BRL49653 supplementation for the remaining 7 days of adipocyte differentiation. Differentiated human visceral adipocytes were serum-starved for 24 h and then treated with increasing concentrations of exendin-4 (25, 50 and 100 nmol/L) (Sigma, St. Louis, MO, USA). In another set of experiments, cells were stimulated with LPS (1000 ng/mL) (Sigma) or under hypoxic conditions mimicked by the divalent transition-metal ion cobalt (CoCl_2_) (100 nmol/mL) (Sigma) for 3 h followed by incubation in the presence or absence of exendin (100 nmol/L) for another 24 h. The adipocyte-conditioned media (ACM) were prepared by collecting the supernatant from differentiated adipocytes after the treatment with the different exendin-4 concentrations. The ACM were centrifuged at 200× *g* for 10 min and the supernatant was collected and stored at −80 °C. In order to assess the concentrations of the pro-inflammatory factors IL-1β, IL-6, IL-8, MCP-1, monocyte chemoattractant protein-1 (MCP-1) and tumor necrosis factor (TNF)-α in the ACM, commercially available ELISA kits (RayBiotech, Inc., Norcross, GA, USA) were used according to the manufacturer’s instructions. The intra- and inter-assay coefficients of variation were <10 and <12%, respectively, for all analysed molecules.

### 2.6. Statistical Analysis

Data are presented as mean ± standard error of the mean (SEM). Differences in the proportion of subjects within groups regarding gender were assessed by the Chi-square test. Differences between groups were assessed by one-way ANOVA followed by Tukey’s or Dunnett’s *post hoc* tests and two-tailed paired or unpaired Student’s *t* tests as appropriate. Differences between groups adjusted for age and sex were determined by analysis of covariance (ANCOVA). Pearson’s correlation coefficients (*r*) were used to analyse the association between variables. The calculations were performed using the SPSS/Windows version 15.0 statistical package (SPSS, Chicago, IL, USA). A *p*-value <0.05 was considered statistically significant.

## 3. Results

### 3.1. Decreased GLP-1 Circulating Levels in Human Obesity-Associated T2D

**Figure 1:**
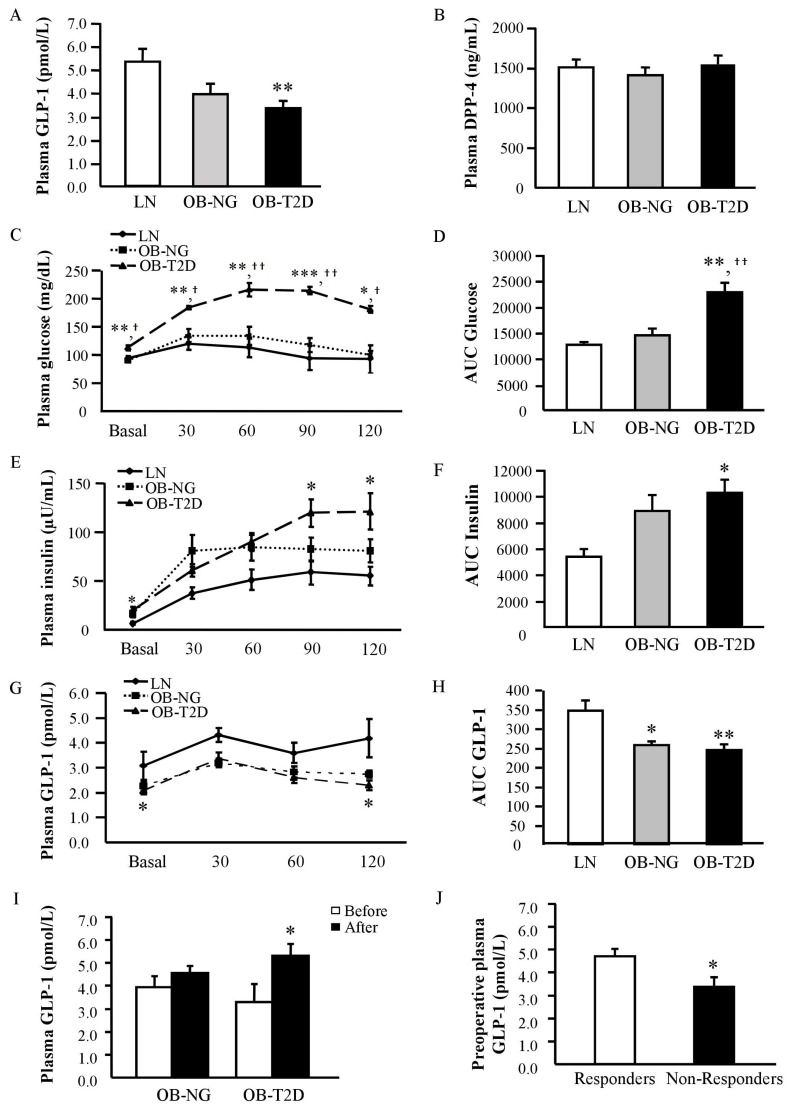
(**A**) Circulating GLP-1 and (**B**) DPP-4 levels in lean (LN) volunteers, obese normoglycemic (NG) and obese patients with type 2 diabetes (T2D). Plasma glucose (**C**), insulin (**E**) and GLP-1 (**G**) concentrations during an OGTT in LN volunteers, obese NG and obese subjects with T2D as well as the area under the curve (AUC) for glucose (**D**), insulin (**F**) and GLP-1 (**H**), respectively. Data represent the mean ± SEM. Differences between groups were analyzed by one-way ANOVA followed by Tukey’s tests. * *p* < 0.05, ** *p* < 0.01, *** *p* < 0.001 vs. LN subjects. ^†^ *p* < 0.05, ^††^ *p* < 0.01 vs. obese NG subjects. (**I**) GLP-1 concentrations in obese patients before and after Roux en-Y gastric bypass (RYGB) and (**J**) comparison of pre-surgical GLP-1 levels in obese patients with T2D classified according to their response to RYGB regarding T2D remission. Bars represent the mean ± SEM. Differences between groups were analyzed by paired or unpaired two-tailed Student’s *t* tests, where appropriate. * *p* < 0.05 vs. pre-surgical values or responder patients.

Baseline characteristics of the subjects enrolled in the study are summarized in Table 1. Markers of adiposity were significantly higher (*p* < 0.001) in patients from both obese groups compared with normal-weight volunteers. As expected, patients with T2D were more insulin resistant than control and obese normoglycemic (NG) volunteers. Adiponectin levels were significantly decreased (*p* < 0.01) in patients with T2D compared to both lean and obese NG volunteers. Notably, individuals with obesity exhibited increased (*p* < 0.01) circulating concentrations of the inflammatory markers CRP, fibrinogen and uric acid. No differences were found in the circulating levels of AST and ALP, but patients with T2D exhibited increased (*p* < 0.01) concentrations of γ-GT. Significant differences in circulating GLP-1 levels among the three experimental groups were observed (*p* < 0.05) to be significantly reduced in obese patients with T2D as compared to lean subjects (Figure 1A). Differences remained statistically significant after the adjustment by age (*p* < 0.05). No differences in circulating levels of DPP-4, an essential enzyme for the initial degradation of GLP-1, were found between the experimental groups (Figure 1B).

We also evaluated glucose (Figure 1C,D), insulin (Figure 1E,F) and GLP-1 (Figure 1G,H) profiles after an OGTT. Plasma glucose concentrations were significantly higher (*p* < 0.01) in obese patients with T2D at all-time points during the OGTT. In this sense, the area under the curve (AUC) for plasma glucose concentration was significantly higher (*p* < 0.01) in T2D patients compared to normal-weight and obese NG volunteers. During the OGTT, serum levels of insulin were gradually increased, with its concentrations at 90 and 120 min being significantly higher (*p* < 0.05) in T2D patients compared to normal-weight volunteers. The AUC for insulin was significantly higher (*p* < 0.05) in obese patients with T2D compared to lean volunteers. While the higher glucose levels of obese T2D patients were obvious from 30 min onwards of the OGTT, their serum insulin concentrations peaked at 90 min. Although the oral glucose load tended to increase plasma levels of GLP-1 mainly at 30 and 120 min in all the experimental groups, obese patients with T2D had lower GLP-1 concentrations than lean volunteers at baseline (3.08 ± 0.55 vs. 2.07 ± 0.13 pmol/L, *p* = 0.019) and after 120 min (4.19 ± 0.78 vs. 2.30 ± 0.08 pmol/L, *p* = 0.004). The decrease in the GLP-1 AUC in obese patients with and without T2D compared to lean volunteers was 30% (*p* < 0.01) and 25 % (*p* < 0.05), respectively.

### 3.2. GLP-1 Circulating Levels in Patients with T2D Increase after RYGB

The effect of RYGB on circulating concentrations of GLP-1 was also analysed. As expected, after an average post-surgical period of one year, patients experienced a significant decrease (*p* < 0.0001) in all anthropometric measurements as well as a significant improvement in the insulin resistance as evidenced by the decrease (*p* < 0.001) in fasting glucose and insulin concentrations together with an increase in the QUICKI index (Table 2). Obese patients also exhibited an amelioration of lipid metabolism supported by a significant reduction (*p* < 0.01) in circulating triglycerides as well as total- and LDL-cholesterol. Importantly, RYGB was associated with an increase (*p* < 0.001) in adiponectin together with a reduction in leptin concentrations (*p* < 0.001). A significant reduction (*p* < 0.05) in the circulating concentrations of IL-6 and IL-18 was observed after bariatric surgery in both groups of obese patients. In this line, a decrease in the circulating levels of MCP-1 and IL-1β was found, however, it was not accompanied by statistically significant changes. Although both obese groups experienced an increase in the circulating concentrations of GLP-1 after surgery, differences were statistically significant in obese patients with T2D, with their levels being restored to similar concentrations as those of lean volunteers (Figure 1I). Interestingly, pre-operative GLP-1 levels were positively associated with weight loss (*r* = 0.64; *p* = 0.002).

### 3.3. Increased Pre-Operative GLP-1 Circulating Levels in Patients with T2D Remission

We addressed whether preoperative levels of GLP-1 differed between patients with T2D that responded or not to RYBG regarding remission of T2D. For both groups of T2D obese patients, follow-up data beyond the three post-operative years were available. Overall, we categorized 47 patients with T2D who underwent RYGB surgery as non-responders (*n* = 16, 34%) and responders (*n* = 31, 66%) (long-term data from 8 patients with T2D submitted to RYBG were not available) (Table 3). As expected, non-responders showed a significantly higher DiaRem score compared to those classified as responders (11.00 ± 1.81 vs. 4.05 ± 0.74; *p* = 0.002). Significantly, individuals who experienced a remission of T2D after RYGB showed higher pre-operative GLP-1 concentrations than non-responder patients (4.77 ± 0.37 vs. 3.60 ± 0.34 pmol/L, *p* < 0.05) (Figure 1J).

### 3.4. Exendin-4 Downregulates the Expression of Pro-Inflammatory Markers in Human Visceral Adipocytes

**Figure 2:**
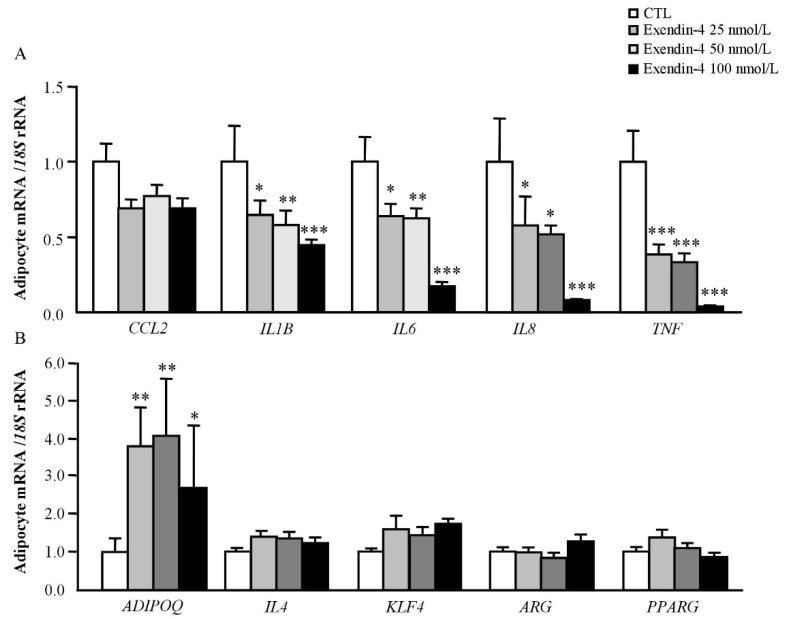
Exendin-4 reduces the expression of pro-inflammatory genes in human visceral adipocytes. (**A**) Gene expression levels of inflammatory factors (monocyte chemoattractant protein-1 (*CCL2*), interleukin (*IL)-1B*, *IL6*, *IL8* and tumour necrosis factor-α (*TNF*)) and (**B**) anti-inflammatory factors (adiponectin (*ADIPOQ*), *IL4*, kruppel-like factor 4 (*KLF4*), arginase 1 (*ARG*) and peroxisome proliferator activated receptor-γ (*PPARG*)] in human visceral adipocytes stimulated with exendin-4 (25, 50 and 100 nmol/L) for 24 h. Gene expression levels in unstimulated cells were assumed to be 1. Values are the mean ± SEM (*n* = 6 per group). Differences between groups were analyzed by one-way ANOVA followed by Dunnett’s tests. * *p* < 0.05, ** *p* < 0.01, *** *p* < 0.001 vs. unstimulated cells.

**Figure 3:**
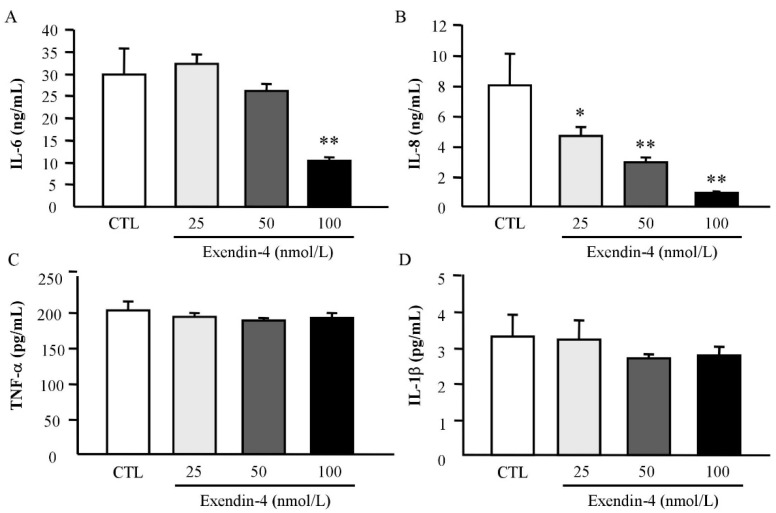
Secreted levels of main inflammatory markers after exendin-4 treatment. (**A**) Interleukin (IL)-6, (**B**) IL-8, (**C**) tumour necrosis factor (TNF)-α and (**D**) IL-1β concentrations in the culture medium of human visceral adipocytes incubated in the presence (25, 50 and 100 nmol/L) or absence of exendin-4 for 24 h. Values are the mean ± SEM (*n* = 6 per group). Differences between groups were analyzed by one-way ANOVA followed by Dunnett’s tests. * *p* < 0.05, ** *p* < 0.01 vs. unstimulated cells.

Since the role of GLP-1 as an anti-inflammatory factor is well-recognized, we assessed whether exendin-4, a GLP-1 receptor agonist, could act as an inhibitor of inflammation in human adipocytes. First, we confirmed that *GLP1R* is expressed in VAT from lean and obese patients observing also an increase in its expression levels in both groups of patients with obesity compared to normal-weight volunteers (Supplementary Material, Figure S1). As shown in Figure 2A, exendin-4 treatment for 24 h significantly downregulated (*p* < 0.05) the mRNA levels of the well-known inflammatory mediators *IL1B*, *IL6*, *IL8* and *TNF* in human visceral adipocytes. Although a decrease in the expression of *CCL2* was also detected, differences were not statistically significant. Conversely, a significant increase (*p* < 0.01) in the expression of the anti-inflammatory gene *ADIPOQ* after exendin-4 treatment was found (Figure 2B). No differences were identified in the expression of *IL4*, *KLF4*, *ARG* and *PPARG* although their mRNA levels tended towards an increase. We also analysed the secretion levels of some major inflammatory mediators in the culture medium after the exendin-4 treatment, finding a significant decrease (*p* < 0.01) in the secretion of IL-6 and IL-8 (Figure 3).

### 3.5. Exendin-4 Inhibits LPS- and Hypoxia-Induced Inflammation in Human Visceral Adipocytes

**Figure 4:**
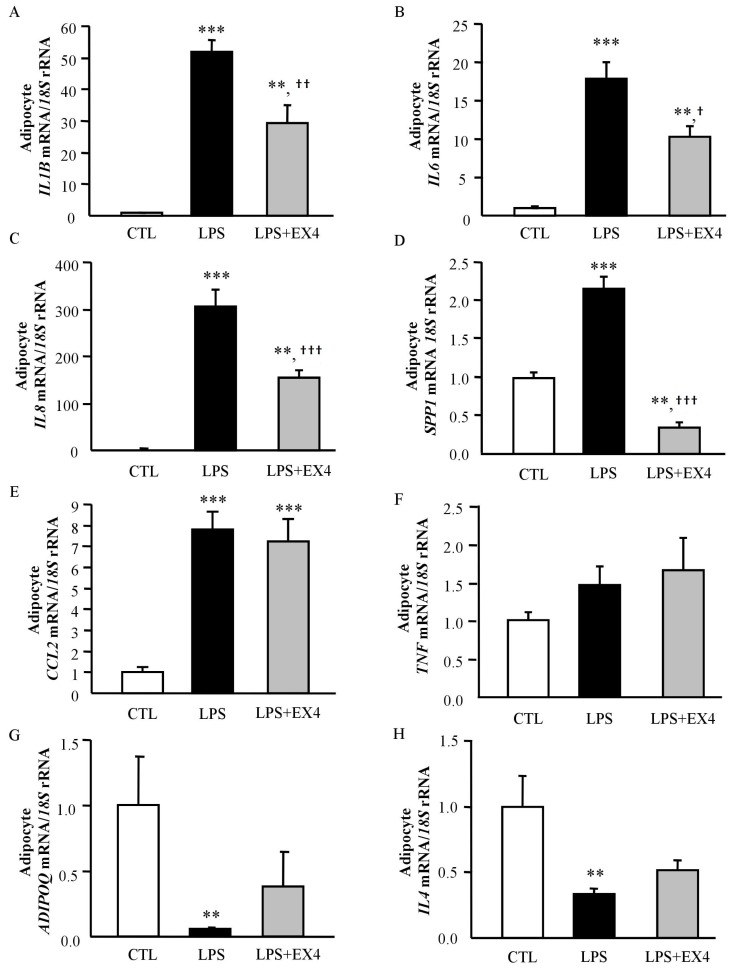
Impact of exendin-4 on LPS-induced inflammation in human visceral adipocytes. Gene expression levels of interleukin (*IL)-1B* (**A**), *IL6* (**B**), *IL8* (**C**), secreted phosphoprotein 1 (osteopontin, *SPP1*) (**D**), monocyte chemoattractant protein-1 (*CCL2*) (**E**), tumour necrosis factor-α (*TNF*) (**F**), adiponectin (*ADIPOQ*) (**G**) and *IL4* (**H**) in human visceral adipocytes incubated with LPS (1000 ng/mL) for 3 h, followed by addition of exendin-4 (100 nmol/L) for another 24 h. Gene expression levels in unstimulated cells were assumed to be 1. Values are the mean ± SEM (*n* = 6 per group). Differences between groups were analyzed by one-way ANOVA followed by Tukey’s tests. ** *p* < 0.01, *** *p* < 0.001 vs. unstimulated cells. ^†^ *p* < 0.05, ^††^ *p* < 0.01, ^†††^ *p* < 0.001 vs. adipocytes stimulated with LPS.

Given the evidence for the anti-inflammatory properties of exendin-4 in adipocytes and the role of LPS as a potent activator of the immune system and a regulator of gut functions, we investigated its effects on LPS-induced inflammation in human adipocytes. As shown in Figure 4, LPS significantly increased (*p* < 0.001) the expression of inflammation-related genes compared to the control group, whereas exendin-4 treatment substantially attenuated the LPS-induced effect by downregulating (*p* < 0.05) the gene expression levels of *IL1B*, *IL6*, *IL8* and *SPP1*. *ADIPOQ* and *IL4* are critical inhibitors of pro-inflammatory targets. In this regard, LPS stimulation significantly reduced *ADIPOQ* and *IL4* gene expression levels, and after exendin-4 treatment their mRNA levels tended to increase although differences did not reach statistical significance. Therefore, these new findings indicate that exendin-4 plays an important role in blocking LPS-induced inflammation in human visceral adipocytes. 

**Figure 5:**
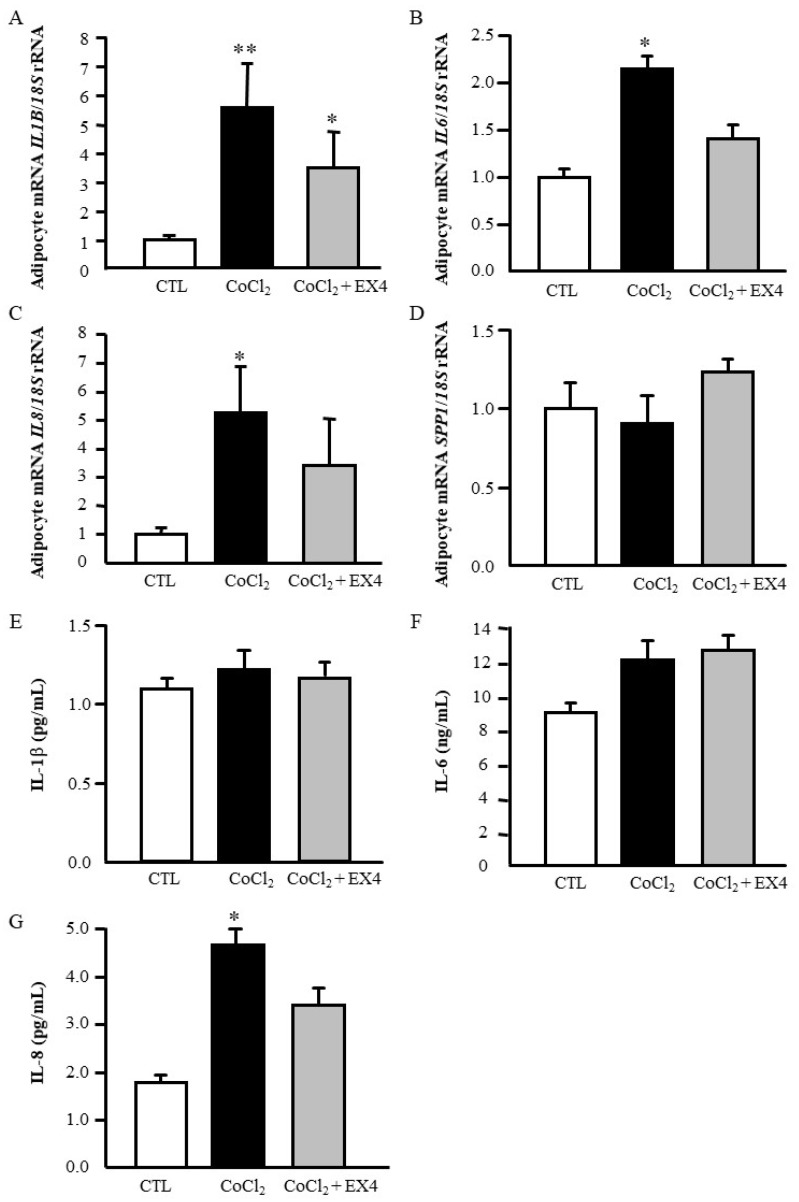
Effects of exendin-4 on hypoxia-induced inflammation in human visceral adipocytes. Gene expression levels (**A**–**D**) and secreted concentrations (**E**–**G**) of the main inflammatory factors in human visceral adipocytes incubated with CoCl_2_ (100 ng/mL) for 3 h followed by the addition of 100 ng/mL exendin-4 for another 24 h. Gene expression levels in unstimulated cells were assumed to be 1. Values are the mean ± SEM (*n* = 6 per group). Differences between groups were analysed by one-way ANOVA followed by Tukey’s tests. * *p* < 0.05, ** *p* < 0.01 vs. unstimulated cells. ^†^ *p* < 0.05 vs. adipocytes stimulated with CoCl_2_.

We found that CoCl_2_ increased (*p* < 0.05) the mRNA levels of *IL1B*, *IL6* and *IL8*, and the treatment with exendin-4 reduced the gene expression levels of these pro-inflammatory factors (Figure 5A–D). In this sense, the treatment with CoCl_2_ promoted an increase (*p* < 0.05) of IL-8 secretion into the culture medium with exendin-4 inhibiting its release. No differences were found in the release of IL-1β and IL-6 after the treatment with exendin-4 (Figure 5E–G). 

## 4. Discussion

Available medical and lifestyle interventions for obesity and its multiple comorbidities have shown modest efficacy and have also been linked to adverse responses and health risks [26]. Surgical management is widely considered the most effective treatment for severe obesity, being associated with a significant health improvement and a reduction of mortality [3,26]. Recently, the incretin GLP-1 and different therapeutic strategies based on the activation of its receptor GLP-1R have been shown to modulate glucose homeostasis as well as body weight and fat mass in animal models suggesting GLP-1 as a possible marker of treatment outcome [11,12]. In this regard, the present study was designed to determine whether glucose homeostasis after RYGB might be related to GLP-1 signalling as well as its possible link with the regulation of adipocyte inflammation. The major finding of our investigation is that reduced pre-operative GLP-1 circulating levels in obese patients with T2D are associated with a worse response in the remission of T2D after RYGB. Consistently, we also found a decrease in the GLP-1 AUC in obese patients after the OGTT and an increase in circulating GLP-1 levels after RYGB in patients with T2D. In addition, we further unveiled an important role of exendin-4, a GLP-1R agonist, in the inhibition of inflammation in human adipocytes.

GLP-1 participates in the stimulation of postprandial insulin release in lean subjects [20], but this incretin response is impaired among subsets of populations of patients with T2D, with some meta-analyses finding an unaltered GLP-1 release [27] and other studies revealing reduced [28] or even increased [29] concentrations of GLP-1 in patients with T2D. These controversial results may be explained by different confounding factors that determine GLP-1 release in patients with T2D including that these patients tended to be older, exhibited increased levels of glucagon and fasting free fatty acids levels as well as differences in assays or in diabetes duration [27]. In this context, our study provided evidence for reduced basal GLP-1 levels in patients with T2D. Our results also showed that the GLP-1 response to an OGTT is reduced in obese patients with and without T2D. In this sense, a lower GLP-1 response in prediabetic or even obese patients has been previously observed [28]. The main differences in GLP-1 levels were after 120 min, suggesting a reduced late response in patients with T2D. In agreement with previous data [6], RYGB induced an increase in GLP-1 levels in patients with T2D. Diverse mechanisms have been proposed including (i) the associated changes in the gastrointestinal anatomy after RYGB, involving a quick nutrient delivery to the lower part of the intestinal tract, where most of L-cells are located, and (ii) the intestinal hypertrophy closely associated with the proliferation of L-cells [6,30]. The increased secretion of GLP-1 has been proposed as one mediator of the increased insulin secretion by pancreatic β-cells [31] but GLP-1 also plays an important role in the regulation of glucose homeostasis [32] or body weight and food intake [33] in other organs, including the central nervous system. However, whether GLP-1 is a good predictor for remission of T2D remains uncertain [12,13,14]. Habegger et al. [12] proposed that GLP-1 might have predictive value for glucose metabolism after RYGB in a rat model of diet induced obesity. In this regard, we detected increased pre-operative GLP-1 concentrations in patients with surgery-induced T2D remission.

The half-life of the intact form of GLP-1 is low since it is rapidly metabolized by the enzyme DPP-4 [8]. Different tissues, including the liver and adipose tissue are considered important sources for circulating DPP-4. Although increased DPP-4 activity has been related to obesity and its associated T2D [34], no differences in DPP-4 were found in our studied groups. In this line, DPP-4 activity has been recently correlated with body weight and fat mass, but not glucose control, in mice [35].

Beyond the critical roles of GLP-1 in the regulation of glucose homeostasis, mainly through the regulation of insulin levels, an anti-inflammatory effect on many tissues including pancreatic islets, adipose tissue, liver, kidney or brain has been described contributing to decreasing glucose levels in T2D [15,16]. In fact, *Glp1r*-knockout mice exhibited dysregulation in the expression of anti-inflammatory genes as well as gut microbiota dysbiosis [36]. In addition, the administration of GLP-1 in an obese mice model of diabetes reduced the oxidative stress and the inflammatory state in adipocytes and macrophages, contributing to the improvement in insulin sensitivity [15]. GLP-1 agonists also have anti-inflammatory effects in different cell types. Reportedly, exendin-4 promotes the upregulation of adiponectin levels in 3T3-L1 adipocytes preventing the production of inflammatory adipokines and ameliorating insulin resistance [37]. Since GLP-1 exerts its functions by binding to its receptor GLP-1R, a G protein-coupled receptor that is widely distributed [8], we first confirmed that *GLP1R* was expressed in the VAT from lean and obese volunteers. In addition, we found that *GLP1R* expression was upregulated in patients with T2D. In this line, Vendrell et al. previously addressed the expression of *GLP1R* in AT and its association with the degree of insulin resistance [38]. Although the mechanisms are not defined, it is clear that GLP-1 enhances insulin sensitivity in peripheral tissues [39]. Our results support the relationship between GLP-1 and insulin sensitivity in AT via the regulation of the inflammatory response. We identified that exendin-4 increases gene expression levels of adiponectin and suppresses that of inflammatory cytokines expression as well as their secretion in human visceral adipocytes, which is in accordance with previous results [37]. The treatment of obese mice with an adenovirus to constitutively produce GLP-1 in vivo, decreased the infiltration of pro-inflammatory macrophages into the AT exerting direct anti-inflammatory effects on adipocytes [15]. These data suggest that GLP-1 may also regulate the recruitment of cells from the immune system into specific tissues to protect them from inflammation. We also found that exendin-4 inhibited the LPS- and hypoxia-induced expression of crucial inflammatory markers in human adipocytes, strengthening the hypothesis that GLP-1 reduces the inflammatory response, potentially contributing to the improvement of insulin sensitivity. In this line, the treatment of human islets with exendin-4 prevented the expression of important inflammatory molecules and, together with the overexpression of CREB, was able to protect the islets to be transplanted into streptozotocin-induced diabetic mice [40].

Taken together, our results indicate that pre-operative GLP-1 represents a biomarker of glucose responsiveness after RYGB and that GLP-1 is involved in limiting VAT inflammation via the downregulation of crucial inflammatory factors. However, the participation of other factors that change following bariatric surgery and also have an impact on glucose homeostasis and inflammation like serum amyloid A, leptin, fibroblast growth factors and caveolin-1, among others, should not be disregarded [41,42,43,44,45]. Thus, further studies are warranted to unravel the underlying mechanisms harnessed by GLP-1 to improve glucose responsiveness after RYGB.

## Figures and Tables

**Table 1:** Anthropometric and biochemical characteristics of subjects included in the study.

	Lean	Obese NG	Obese T2D
*n* (males, females)	30 (12, 18)	30 (12, 18)	33 (16, 17)
Age (years)	46 ± 2	39 ± 2	51 ± 2 ^†^
BMI (kg/m^2^)	23.0 ± 0.2	46.0 ± 1.6 ***	43.1 ± 1.2 ***
Body fat (%)	29.1 ± 1.8	52.1 ± 1.1 ***	48.7 ± 1.4 ***
Waist (cm)	83 ± 1	130 ± 3 ***	127 ± 2 ***
Waist-to-hip ratio	0.87 ± 0.01	0.96 ± 0.02 ***	1.00 ± 0.01 ***
SBP (mm Hg)	109 ± 2	126 ± 3 ***	129 ± 3 ***
DBP (mm Hg)	67 ± 1	80 ± 2 ***	80 ± 2 ***
Fasting glucose (mg/dL)	92 ± 2	90 ± 2	151 ± 11 ***^,^^†††^
Glucose 2-h after OGTT (mg/dL)	98 ± 8	119 ± 4	220 ± 21 ***^,†††^
Fasting insulin (μU/mL)	6.1 ± 0.8	16.8 ± 3.8	28.2 ± 6.1 **
HOMA	1.4 ± 0.2	3.8 ± 0.9	8.2 ± 1.0 ***^,^^†††^
QUICKI	0.375 ± 0.010	0.340 ± 0.010	0.296 ± 0.006 ***^,^^†††^
Triacylglycerol (mg/dL)	80 ± 7	101 ± 6	156 ± 11 ***^,^^††^
Total cholesterol (mg/dL)	180 ± 6	199 ± 9	179 ± 6
LDL-cholesterol (mg/dL)	103 ± 5	128 ± 7 *	104 ± 6 ^†^
HDL-cholesterol (mg/dL)	59 ± 2	50 ± 3 *	44 ± 2 ***
Leptin (ng/mL)	11.4 ± 3.0	50.2 ± 4.0 ***	44.7 ± 5.2 ***
Adiponectin (µg/mL)	13.1 ± 1.9	11.6 ± 1.6	6.7 ± 0.6 ***^,^^††^
Uric acid (mg/dL)	4.5 ± 0.2	5.7 ± 0.3 *	5.8 ± 0.3 **
Creatinine (mg/dL)	0.8 ± 0.03	0.78 ± 0.03	0.82 ± 0.03
CRP (mg/L)	4.81 ± 1.74	9.23 ± 1.82 *	8.27 ± 1.43 *
Fibrinogen (mg/dL)	279 ± 18	394 ± 17 **	370 ± 20 *
von Willebrand factor (%)	108 ± 4	115.1 ± 11.4	148.5 ± 9.2
Homocysteine (μmol/L)	9.7 ± 2.4	10.2 ± 1.7	10.4 ± 1.0
AST (IU/L)	14 ± 1	17 ± 3	16 ± 1
ALT (IU/L)	17 ± 2	24 ± 3 *	23 ± 2
AST/ALT	0.96 ± 0.08	0.76 ± 0.06 *	0.73 ± 0.03 *
ALP (IU/L)	82 ± 6	75 ± 6	67 ± 6
γ-GT (IU/L)	14 ± 1	17 ± 2	33 ± 5 ***^,^^††^

ALP, alkaline phosphatase; ALT, alanine aminotransferase; AST, aspartate aminotransferase; BMI, body mass index; CRP, C-reactive protein; DBP, diastolic blood pressure; γ-GT, γ-glutamyltransferase; HOMA, homeostasis model assessment; NG, normoglycemic; OGTT, oral glucose tolerance test; QUICKI, quantitative insulin sensitivity check index; SBP, systolic blood pressure; T2D, type 2 diabetes. Data are mean ± SEM. Differences between groups were analyzed by one-way ANOVA followed by Tukey’s *post hoc* tests. * *p* < 0.05, ** *p* < 0.01 and *** *p* < 0.001 vs. lean. ^†^ *p* < 0.05, ^††^ *p* < 0.01 and ^†††^ *p* < 0.001 vs. obese NG.

**Table 2:** Effects of weight loss in obese patients after Roux-en-Y gastric bypass (RYGB).

	Obese NG Before Surgery	Obese NG After Surgery	Obese T2D Before Surgery	Obese T2D After Surgery
*n* (males, females)	22 (6, 16)	22 (6, 16)	55 (22, 33)	55 (22, 33)
BMI (kg/m^2^)	44.0 ± 1.9	30.8 ± 1.7 ***	45.7 ± 1.0	35.2 ± 1.0 ***
Body fat (%)	52.3 ± 1.0	38.9 ± 2.6 ***	51.6 ± 1.0	43.0 ± 1.5 ***
Waist (cm)	123 ± 4	98 ± 4 ***	130 ± 2	110 ± 2 ***
Waist-to-hip ratio	0.94 ± 0.02	0.90 ± 0.02 *	0.99 ± 0.01	0.96 ± 0.01 **
SBP (mm Hg)	123 ± 3	111 ± 3 ***	130 ± 2	118 ± 2 **
DBP (mm Hg)	78 ± 2	70 ± 2 **	82 ± 1	73 ± 1 *
Fasting glucose (mg/dL)	91 ± 2	87 ± 2 *	122 ± 5	104 ± 5 **
Fasting insulin (μU/mL)	15.1 ± 5.2	6.8 ± 1.3	22.2 ± 1.8	10.2 ± 1.0 **
HOMA	3.7 ± 1.3	1.6 ± 0.3	6.5 ± 0.5	2.5 ± 0.3 ***
QUICKI	0.35 ± 0.01	0.39 ± 0.01 **	0.30 ± 0.01	0.35 ± 0.01 ***
HbA1c (%)	-	-	7.9 ± 0.4	6.7 ± 0.3 **
Triacylglycerol (mg/dL)	101 ± 9	72 ± 7 **	142 ± 13	94 ± 8 ***
Total cholesterol (mg/dL)	201 ± 12	152 ± 7 ***	185 ± 7	157 ± 5 **
LDL-cholesterol (mg/dL)	129 ± 10	83 ± 6 ***	110 ± 6	88 ± 4 **
HDL-cholesterol (mg/dL)	51 ± 6	55 ± 3	46 ± 2	51 ± 2 *
Leptin (ng/mL)	49.1 ± 5.2	12.5 ± 3.1 ***	48.7 ± 4.6	17.7 ± 1.9 ***
Adiponectin	11.6 ± 1.3	16.9 ± 1.3 ***	7.2 ± 0.4	12.0 ± 0.7 ***
Uric acid (mg/dL)	5.5 ± 0.3	4.8 ± 0.3 **	5.9 ± 0.2	5.3 ± 0.2 *
Creatinine (mg/dL)	0.7 ± 0.1	0.7 ± 0.2	0.8 ± 0.1	1.0 ± 0.2 *
CRP (mg/L)	10.23 ± 3.20	4.96 ± 0.30 *	7.91 ± 1.99	2.48 ± 0.76 *
Fibrinogen (mg/dL)	394 ± 23	342 ± 20 **	364 ± 17	350 ± 16
von Willebrand factor (%)	97 ± 18	91 ± 15	155 ± 13	127 ± 12 *
Homocysteine (μmol/L)	14.1 ± 5.8	10.6 ± 2.8	10.1 ± 0.9	9.9 ± 0.7
AST (IU/L)	17 ± 4	14 ± 1	15 ± 1	18 ± 1
ALT (IU/L)	24 ± 4	16 ± 2	23 ± 2	26 ± 3
AST/ALT	0.74 ± 0.07	0.90 ± 0.04 **	0.75 ± 0.04	0.80 ± 0.04
ALP (IU/L)	78 ± 8	61 ± 5 **	70 ± 5	72 ± 5
γ-GT (IU/L)	17 ± 2	10 ± 2 ***	31 ± 3	18 ± 1 ***
IL-1β (pg/mL)	1.57 ± 0.18	1.41 ± 0.07	1.61 ± 0.10	1.42 ± 0.08
IL-6 (pg/mL)	19.8 ± 8.2	8.5 ± 0.4 *	19.1 ± 2.6	11.1 ± 0.8 **
IL-18 (pg/mL)	126.8 ± 9.2	99.4 ± 7.6 *	122.9 ± 6.7	112.5 ± 6.3 *
MCP-1 (pg/mL)	221 ± 32	198 ± 21	247 ± 25	234 ± 20

ALP, alkaline phosphatase; ALT, alanine aminotransferase; AST, aspartate aminotransferase; BMI, body mass index; CRP, C-reactive protein; DBP, diastolic blood pressure; γ-GT, γ-glutamyltransferase; HbA1c, glycated hemoglobin; HOMA, homeostasis model assessment; IL, interleukin; MCP-1, monocyte chemoattractant protein; NG, normoglycemic, QUICKI, quantitative insulin sensitivity check index; SBP, systolic blood pressure; T2D, type 2 diabetes. Data are mean ± SEM. Differences between groups were analyzed by two-paired Student *t* tests. * *p* < 0.05, ** *p* < 0.01 and *** *p* < 0.001 vs. before surgery.

**Table 3:** Anthropometric and biochemical characteristics of obese patients with T2D classified as responders and non-responders one year after RYGB.

	T2D-Responders	T2D-Non-Responders
*n* (males, females)	31 (16, 15)	16 (8, 8)
DiaRem Score	4.05 ± 0.74	11.00 ± 1.81 **
Insulin treatment (%)	6.5%	31.5%
Other T2D treatment (%)	19.4%	87.5%
Age (years)	48 ± 2	52 ± 2
BMI (kg/m^2^)	41.2 ± 1.8	39.3 ± 1.8
Body fat (%)	48.4 ± 1.3	44.8 ± 1.8
Waist (cm)	126 ± 2	121 ± 4
Waist-to-hip ratio	0.99 ± 0.01	0.99 ± 0.02
SBP (mm Hg)	132 ± 3	128 ± 4
DBP (mm Hg)	81 ± 2	77 ± 1
HOMA	7.8 ± 1.3	10.9 ± 2.7
QUICKI	0.296 ± 0.006	0.291 ± 0.014
Triacylglycerol (mg/dL)	126 ± 11	228 ± 39 *
Total cholesterol (mg/dL)	184 ± 7	207 ± 27
LDL-cholesterol (mg/dL)	106 ± 7	97 ± 6
HDL-cholesterol (mg/dL)	49 ± 4	37 ± 3
Uric acid (mg/dL)	5.5 ± 0.3	6.2 ± 0.8
Creatinine (mg/dL)	0.82 ± 0.03	0.97 ± 0.09 *
CRP (mg/L)	0.8. ± 0.1	0.6 ± 0.3
Fibrinogen (mg/dL)	391 ± 26	340 ± 68
von Willebrand factor (%)	135 ± 11	129 ± 21
AST (UI/L)	16 ± 1	20 ± 4
ALT (UI/L)	27 ± 3	31 ± 7
ALP (UI/L)	78 ± 9	69 ± 14
γ-GT (UI/L)	24 ± 3	48 ± 18 ***

ALP, alkaline phosphatase; ALT, alanine aminotransferase; AST, aspartate aminotransferase; BMI, body mass index; CRP, C-reactive protein; DBP, diastolic blood pressure; γ-GT, γ-glutamyltransferase; HOMA, homeostasis model assessment; QUICKI, quantitative insulin sensitivity check index; SBP, systolic blood pressure; Data are mean ± SEM. Differences between groups were analyzed by *t*-Student tests. * *p* < 0.05, ** *p* < 0.01 and *** *p* < 0.001 vs. Responders.

## Supplementary Materials

The following are available online at https://www.mdpi.com/2077-0383/8/4/479/s1, Figure S1: Gene expression levels of GLP1R in lean, obese NG and obese patients with T2D, Table S1: Sequences of the primers and TaqMan^®^ probes.

## Abbreviations

ADIOPQ	adiponectin
ALP	alkaline phosphatase
ALT	alanine aminotransferase
ARG1	arginase 1
AST	aspartate aminotransferase
BMI	body mass index
CCL2	monocyte chemoattractant protein-1
CRP	C-reactive protein
DBP	diastolic blood pressure
γ-GT	γ-glutamyltransferase
GLP-1	glucagon-like peptide-1
HOMA	homeostasis model assessment
IL	interleukin
KLF4	kruppel-like factor 4
LPS	lipopolysaccharide
NG	normoglycemic
OGTT	oral glucose tolerance test
PARG	peroxisome proliferator-activated receptor-γ
QUICKI	quantitative insulin sensitivity check index
RYGB	Roux-en-Y gastric bypass
SBP	systolic blood pressure;
SVFC	stroma-vascular fraction cells;
TNF	tumour necrosis factor-α
T2D	type 2 diabetes
VAT	visceral adipose tissue
