# Supplementary material for: GLP-1 Limits Adipocyte Inflammation and Its Low Circulating Pre-Operative Concentrations Predict Worse Type 2 Diabetes Remission after Bariatric Surgery in Obese Patients
Source: J Clin Med. 2019 Apr 9;8(4):479. doi: 10.3390/jcm8040479 (PMC6518381; doi:10.3390/jcm8040479)
Supplement: Supplementary file 1 [file jcm-08-00479-s001.pdf]

Supplemental Table 1. Sequences of the primers and TaqMan® probes

Gene (GenBank accession)	Oligonucleotide sequence (5'-3')
ADIPOQ (NM_001177800)	
Forward	GGAGATCCAGGTCTTATTGGTCCTA
Reverse	CCTTGGATTCCCGGAAAGC
TaqMan® Probe	FAM-ACATCGGTGAAACCGGAGTACCCGG-TAMRA
ARG1 (NM_000045)	
Forward	TACTAGGAAGAAAGAAAAGGCCAATTC
Reverse	GTAGCCCTGTTTTGTAGATTTCTTCTGT
TaqMan® Probe	FAM-ACCCATCTTTCACACCAGCTACTGGCACA-TAMRA
CCL2 (NM_002982)	
Forward	GCTCATAGCAGCCACCTTCATT
Reverse	TCTGCACTGAGATCTTCCTATTGGT
TaqMan® Probe	FAM-TCGCTCAGCCAGATGCAATCAATGC-TAMRA
IL1B (NM_000576)	
Forward	CAGTGGCAATGAGGATGACTTG
Reverse	GTAGTGGTGGTCGGAGATTCGTA
TaqMan® Probe	FAM-TGGCCCTAAACAGATGAAGTGCTCCTTCC-TAMRA
IL4 (NM_000589)	
Forward	GCCTCCAAGAACACAACTGAGAA
Reverse	TGTCGAGCCGTTTCAGGAAT
TaqMan® Probe	FAM-CTGCGACTGTGCTCCGGCAGTTCTA-TAMRA
IL6 (NM_000600)	
Forward	GCCCTGAGAAAGGAGACATGTAAC
Reverse	ATCCATCTTTTTCAGCCATCTTTG
TaqMan® Probe	FAM-AGGCACTGGCAGAAAACAACCTGAACC-TAMRA
IL8 (NM_000584.3)	
Forward	ACCTTTCCACCCCAAATTTATCA
Reverse	TTCTCAGCCCTCTTCAAAAACTTC
TaqMan® Probe	FAM-CCACACTGCGCCAACACAGAAATTATTGTA-TAMRA
KLF4 (NM_001314052.1)	
Forward	ACCTACACAAAGAGTTCCCATCTCA
Reverse	GTTTACGGTAGTGCCTGGTCAGTT
TaqMan® Probe	FAM-CCTGCGAACCCACACAGGTGAGAAA-TAMRA
PPARG (NM_005037)	
Forward	GGGATGTCTCATAATGCCATCAG
Reverse	CCGCCAACAGCTTCTCCTT
TaqMan® Probe	FAM-TTTGGGCGGATGCCACAGGC-TAMRA
TNF (NM_000594)	
Forward	CCCCAGGGACCTCTCTCTAATC
Reverse	ACATGGGCTACAGGCTTGTCA

TaqMan® Probe	FAM-CCTCTGGCCCAGGCAGTCAGATCAT-TAMRA

ADIOPQ, adiponectin; *ARG1*, arginase 1; *CCL2*, monocyte chemoattractant protein-1; *IL*, interleukin; *KLF4*, kruppel-like factor 4; *PPARG*, peroxisome proliferator-activated receptor-γ; *TNF*, tumor necrosis factor-α.

Supplemental figure

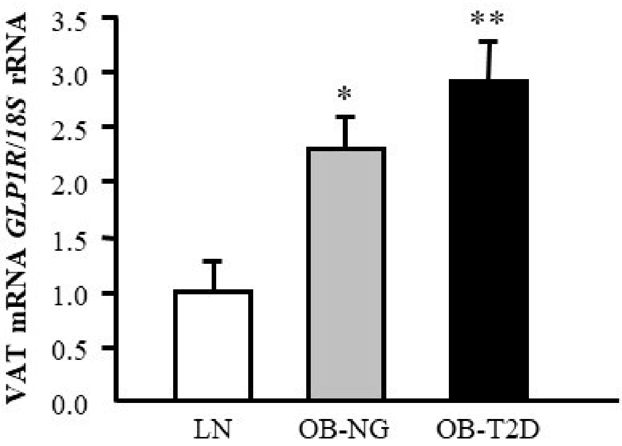

Supplemental Figure 1. Gene expression levels of *GLP1R* in lean, obese NG and obese patients with T2D. Bar graphs show the mRNA levels of *GLP1R* in visceral adipose tissue from lean (LN) volunteers, obese normoglycemic (NG) subjects and obese patients with type 2 diabetes (T2D). Bars represent the mean ± SEM. LN was assumed to be 1. Differences between groups were analyzed by one-way ANOVA followed by Tukey's tests. $^{*}P<0.05$, $^{**}P<0.01$ *vs* LN subjects.
